# Serum levels of soluble programmed death ligand 1 predict treatment response and progression free survival in multiple myeloma

**DOI:** 10.18632/oncotarget.5682

**Published:** 2015-10-16

**Authors:** Liang Wang, Hua Wang, Hao Chen, Wei-da Wang, Xiao-qin Chen, Qi-rong Geng, Zhong-jun Xia, Yue Lu

**Affiliations:** ^1^ State Key Laboratory of Oncology in South China, Collaborative Innovation Center for Cancer Medicine, Guangzhou, Guangdong, 510060, People's Republic of China; ^2^ Department of Hematologic Oncology, Sun Yat-sen University Cancer Center, Guangzhou, Guangdong, 510060, People's Republic of China; ^3^ Department of Clinical Laboratory, Sun Yat-sen University Cancer Center, Guangzhou, Guangdong, 510060, People's Republic of China

**Keywords:** multiple myeloma, programmed death-ligand 1, immune checkpoint, prognosis, biomarker

## Abstract

Immune checkpoint signaling plays an important role in immunosuppression in multiple myeloma (MM). Blood levels of soluble programmed death-ligand 1 (sPD-L1), a checkpoint-relevant protein, might predict treatment response and survival outcomes in MM patients. We used an enzyme-linked immunosorbent assay to measure serum sPD-L1 levels in 81 newly diagnosed MM patients. We found that myeloma patients had higher sPD-L1 concentrations than healthy controls. The best sPD-L1 cutoff value for predicting disease progression risk was 2.783 ng/mL. The overall response rate to treatment was higher in low sPD-L1 patients than in high sPD-L1 patients. The 3-year progression free survival (PFS) and overall survival (OS) rates for all patients were 16% and 64%, respectively. Multivariate survival analysis including Eastern Cooperative Oncology Group performance status score, treatment response, and sPD-L1 level showed that a less than partial treatment response (PR) and higher sPD-L1 levels (>2.783 ng/ml) were independent prognostic factors for shorter PFS; neither factor was predictive of OS. The serum sPD-L1 level is a valuable biomarker for predicting treatment response and an independent prognostic factor for PFS. PD-1/PD-L1 blockade may be a promising novel immune-based therapeutic strategy in MM.

## INTRODUCTION

Multiple myeloma (MM) is a fatal plasma cell malignancy that mainly affects older individuals [1]. Achieving a complete response to treatment is crucial for long-term control of MM [2–4]. The advent of novel proteasome inhibitors and immunomodulatory drugs has improved response rates and progression-free survival (PFS) [5]. However, MM remains incurable, and nearly all patients eventually relapse and succumb to the disease. Drug resistance is a major challenge in treating relapses of MM. Thus, alternative treatment methods that target novel mechanisms to overcome drug resistance are an area of active research. Furthermore, biomarkers that can predict patients' drug response would be helpful in choosing optimal treatment strategies for MM.

Tumor-infiltrating lymphocytes (TILs) are an important component of the immune response to tumors. However, in patients with various types of cancer, lymphocyte activity is inhibited [6]. The programmed death 1 (PD-1) receptor protein acts as an immune checkpoint, suppressing T-cell mediated immune response [7]. PD-1 is typically expressed by activated lymphocytes, and it has two ligands: PD ligand 1 (PD-L1) and PD-L2. Ligand binding down-regulates antigen-stimulated lymphocyte proliferation and cytokine production, ultimately resulting in lymphocyte ‘exhaustion’ and the induction of immune invasion [7–9]. Inhibition of these checkpoints can restore immune activity against cancer cells. Recent clinical trials show that PD-1–blocking antibodies can enhance immunity in solid tumors and several hematologic malignancies, resulting in durable clinical responses [10–13].

Previous studies have found that myeloma cells express PD-L1, and proinflammatory signals increase this expression [14–16]. Gorgun *et al*. [17] demonstrated that PD-1/PD-L1 blockade reduced bone marrow stroma cell (BMSC)-induced tumor growth. Furthermore, lenalidomide significantly reduced PD-L1 expression in MM cells, and combining lenalidomide with PD-1/PD-L1 blockade further decreased BMSC-induced MM growth. Thus, immune checkpoint signaling plays an important role in promoting tumor growth and suppressing immune response in MM. Targeting checkpoint signaling using PD-1 and PD-L1 blocking antibodies is a promising novel immune-based therapeutic strategy for MM. Rossille *et al*. [18] recently found that the soluble PD-L1 (sPD-L1) concentration in blood could predict overall survival and treatment response in diffuse large B cell lymphoma (DLBCL). In this study, we investigated the expression of sPD-L1 in MM patients, and explored the value of sPD-L1 levels in predicting treatment response.

## RESULTS

### Patients' characteristics and correlation with sPD-L1 level

**Figure 1:**
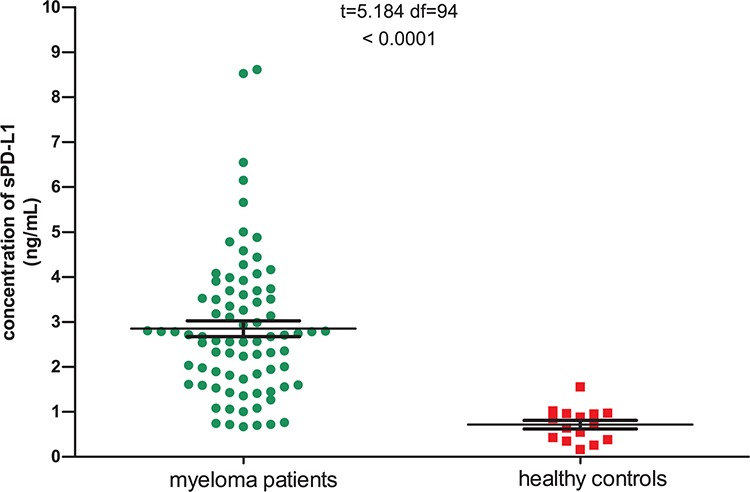
Serum sPD-L1 levels in patients with multiple myeloma and healthy controls The mean concentration of sPD-L1 for 81 myeloma patients was 2.851 ng/ml, significantly higher than that of 15 healthy controls (0.716 ng/mL, *p* < 0.0001).

As is shown in Table 1, most patients (77.8%) were male, and more than half (55.6%) were under than 60 years old. The stages were balanced among stage I (32.1%), II (38.3%) and III (29.6%). About 40% of patients had an Eastern Cooperative Oncology Group (ECOG) score greater than 2 due to myeloma-related bone pain or disability. The mean concentration of sPD-L1 for myeloma patients was 2.851 ng/mL, much higher than that of healthy controls (0.716 ng/mL, *p* < 0.0001, Figure 1). There was no significant correlation between sPD-L1 level and gender, age, International staging system (ISS) stage, lactate dehydrogenase (LDH) level, renal function, or treatment regimens (*p* > 0.05). However, patients with poor performance status (PS) had higher sPD-L1 levels (*p* = 0.005).

**Table 1:** Patients' characteristics and sPD-L1 level

Parameters		*N* = 81(%)	sPD-L1 level (ng/mL) (mean ± SD)	*P* value
Gender	Male	63(78%)	2.885 ± 1.704	0.716
	Female	18(22%)	2.730 ± 1.069	
Age	>60	36(44%)	2.924 ± 1.556	0.714
	= <60	45(56%)	2.793 ± 1.615	
ISS stage	I	26(32%)	2.819 ± 1.247	0.686*
	II	31(39%)	2.987 ± 1.769	
	III	24(29%)	2.709 ± 1.694	0.793**
Serum creatinine level	<2mg/dL	74(91%)	2.810 ± 1.632	0.450
	>=2mg/dL	7(9%)	3.286 ± 0.810	
ECOG PS score	0–2	49(60%)	2.463 ± 1.300	0.005
	>2	32(40%)	3.446 ± 1.796	
LDH level	Normal	65(80%)	2.763 ± 1.503	0.316
	Elevated	16(20%)	3.208 ± 1.874	
Treatment regimen	Bortezomib-based	26(32%)	2.565 ± 1.648	0.265
	Old-drugs-based	55(68%)	2.986 ± 1.544	
Treatment response	CR+PR	42(52%)	2.572 ± 1.556	0.099
	Less than PR	39(48%)	3.152 ± 1.571	
Disease progression	Yes	51(63%)	3.264 ± 1.736	<0.0001
	No	30(37%)	2.149 ± 0.941	

Abbreviations: sPD-L1, soluble programmed death-ligand 1; ISS, international staging system; ECOG, Eastern Cooperative Oncology Group; PS, performance status; LDH, lactate dehydrogenase; CR, complete response; PR, partial response; SD, standard deviation

* stage I vs. stage II

** stage I vs. stage III

### Treatment response and correlation with sPD-L1 level

**Figure 2:**
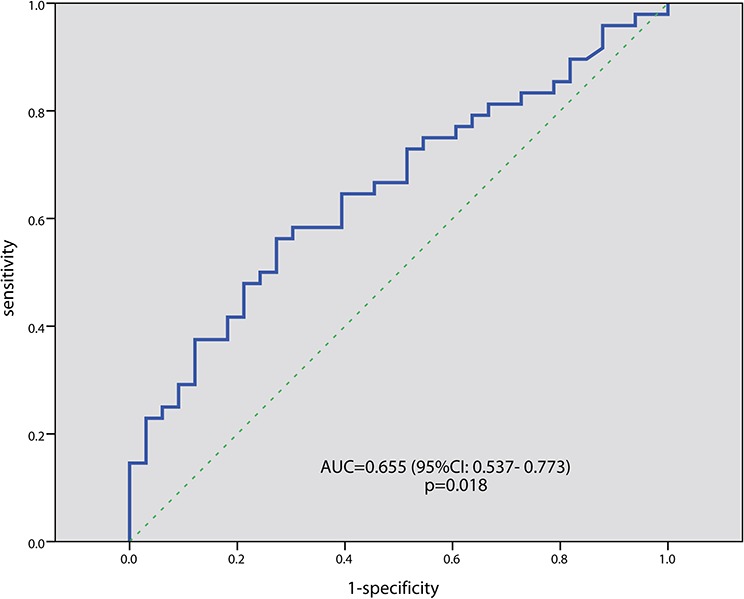
ROC curve analysis for the optimal cut-off point of serum sPD-L1 concentration The most discriminative cut-off value for sPD-L1 was 0.273 ng/mL with an AUC value of 0.655 (*p* = 0.018). The sensitivity and specificity were 56.3% and 72.7%, respectively.

After at least 4 cycles of treatment, 12 patients (15%) showed a complete response (CR), and 42 patients (51.9%) showed at least a partial response (PR). As is shown in Table 1, patients with less than PRs tended to have higher sPD-L1 levels than those with at least a PR (*p* = 0.099). As is shown in Figure 2, the best cutoff value defined by ROC curve for sPD-L1 in predicting high risk for disease progression is 2.783 ng/mL, with an AUC of 0.655 (*p* = 0.018). According to this cutoff value, 36 patients (44.4%) were classified as the high sPD-L1 level group (>2.783 ng/mL), and the remaining 45 patients (55.6%) were classified as the low sPD-L1 level group (= < 2.783 ng/mL). The CR rate in the high sPD-L1 group was 8.3% (3 of 36 patients), while in the low sPD-L1 group it was 20.0% (*p* = 0.249). The overall response rate (ORR, including CR and PR) was 66.7% in low sPD-L1 group, significantly higher than the high sPD-L1 group (33.3%, *p* = 0.006). The ORR was significantly higher in patients treated with novel drug-based regimens than those with older drug-based regimens (69.2% vs. 43.6%, *p* = 0.036).

### Survival analysis

**Figure 3:**
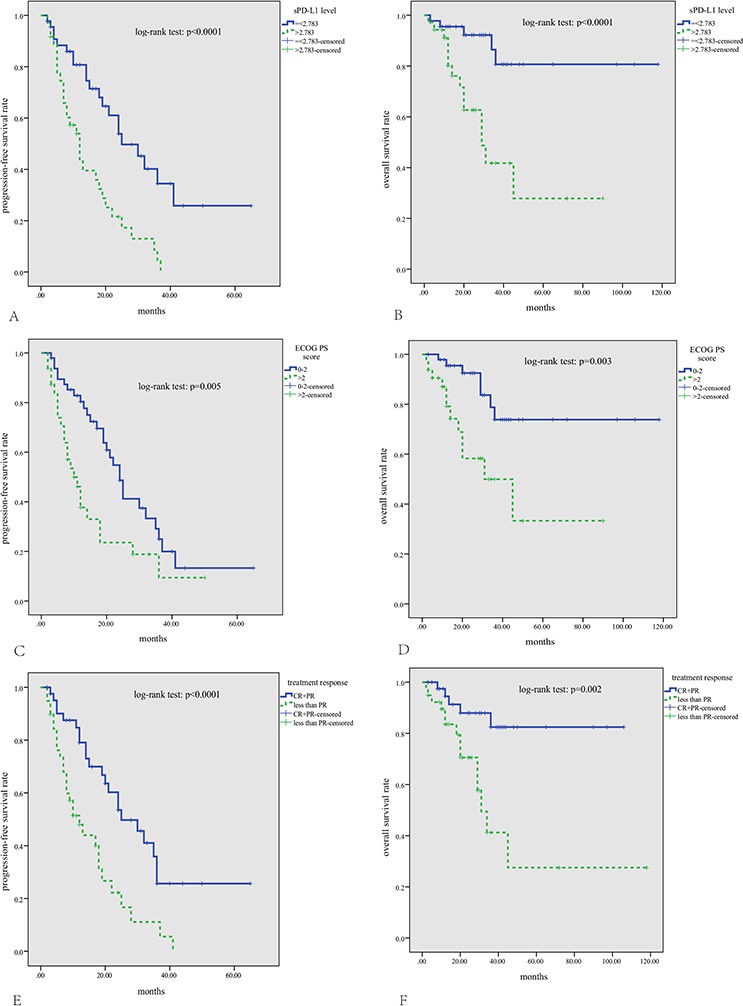
Kaplan-Meier survival analysis for all patients with multiple myeloma Patients with lower sPD-L1 levels (= < 2.783 ng/mL) **A, B.** good ECOG PS scores (0–2) **C, D.** and good treatment response (CR+PR) **E, F.** had significantly longer PFS and OS (*p* < 0.05).

At a median follow-up time of 38 months (range 2–69 months), disease progression occurred in 51 patients at a median of 12 months (range 2–41 months), and 19 patients died of tumor progression at a median of 18 months (range 2–45 months). The 3-year PFS and OS rates were 16% and 64%, respectively. As is shown in Figure 3 and Table 2, patients with lower sPD-L1 levels (= < 2.783 ng/ml), good ECOG PS score (0–2), and good treatment response (CR+PR) had higher PFS and OS rates (*p* < 0.05). However, age, ISS stage, LDH level, and different treatment regimens did not affect long-term outcomes (*p* > 0.05). A multivariate survival analysis including ECOG PS score, treatment response, and sPD-L1 level showed that both less than PR to treatment and higher sPD-L1 levels (>2.783 ng/ml) were independent prognostic factors for lower PFS, but neither was predictive of OS.

**Table 2:** Univariate and multivariate survival analysis

Parameters	PFS			OS		
	Univariate analysis	Multivariate analysis		Univariate analysis	Multivariate analysis	
	*P* value	HR (95%CI)	*P* value	*P* value	HR (95%CI)	*P* value
Age	0.544			0.523		
Gender	0.141			0.381		
Stage	0.838			0.619		
ECOG PS score (>2)	0.005	1.751 (0.977–3.139)	0.060	0.003	2.189 (0.809–5.922)	0.123
LDH level	0.803			0.294		
Treatment regimens	0.737			0.160		
Treatment response (less than PR)	<0.0001	1.959 (1.048–3.663)	0.035	0.002	2.217 (0.696–7.066)	0.178
sPD-L1 level (>2.783 ng/mL)	<0.0001	1.955 (1.029–3.712)	0.041	<0.0001	2.668 (0.818–8.702)	0.104

Abbreviations: PFS, progression-free survival; OS, overall survival; sPD-L1, soluble programmed death-ligand 1; ECOG, Eastern Cooperative Oncology Group; PS, performance status; LDH, lactate dehydrogenase; PR, partial response; HR, hazard ratio.

## DISCUSSION

Blockade of the PD1-PDL1 pathway is a new and promising therapeutic approach in MM. We investigated serum levels of sPD-L1 in a large series of MM patients to identify any correlations with patient characteristics and survival outcomes. We found that serum sPD-L1 concentrations in MM patients were much higher than in normal healthy people. In MM patients, serum sPD-L1 levels were correlated with ECOG PS score, but not with any other clinical feature. However, increased pretreatment serum sPD-L1 levels were associated with poor treatment responses. Furthermore, a Cox regression model including ECOG PS score, treatment response, and sPD-L1 level showed that a higher sPD-L1 level (>2.783 ng/mL) was a noteworthy independent prognostic factor for lower PFS.

In recent years, the roles of PD-1 and PD-L1 in tumor progression and chemotherapy resistance have been extensively studied. Surface PD-L1 expression is high in MM cells [14, 16, 17], and direct interaction between PD-L1 on myeloma cells and PD-1 on T cells induces resistance to anti-myeloma chemotherapy [19]. In this study, we detected significantly higher levels of sPD-L1 in patients with MM compared to healthy controls. Furthermore, higher sPD-L1 levels (>2.783 ng/mL) were correlated with poor treatment response and lower PFS. Serum sPD-L1 concentrations in healthy donors increase with age, suggesting that levels of circulating sPD-L1 are associated with the health of an individual's immune system [20]. However, the sources of sPD-L1 remain unknown. Generally, soluble forms of similar ligands are produced primarily through proteolytic cleavage of membrane-bound proteins such as sB7-H3 [21]. A small portion is also produced by translation of alternatively spliced mRNA, as is the case for sCTLA-4 [22]. sPD-L1 was detectable in supernatants from membrane PD-L1 +, but not PD-L1 -, cell lines, indicating that PD-L1 expressed on the cell surface may be a source of sPD-L1 [20]. It is possible that soluble and membrane-bound PD-L1 bind to PD-1 similarly; thus, sPD-L1 may play an important role in the regulation of immune activity.

Whether non-MM cells produce sPD-L1 remains unknown. Rossille *et al*. [18] did not find an association between plasma sPD-L1 levels and tumor PD-L1 expression in DLBCL patients, suggesting that non-malignant cells in the tumor microenvironment can produce sPD-L1 in response to pro-inflammatory cytokines [23, 24]. We previously measured sPD-L1 levels in supernatants from two myeloma cell lines (U266 and RPMI8226) and found that both lines produced sPD-L1 (0.533 ng/ml and 0.443 ng/mL, respectively). Moreover, the sPD-L1 level could be increased by co-culturing the cells with pro-inflammatory cytokines (IL-6 and IFN-γ) (see Supplemental Figure-1A and 1B). Since PD-1/PD-L1 signaling promotes tumor growth while inhibiting anti-tumor immune responses, the correlation between sPD-L1 and disease progression is not surprising. More interestingly, the lack of correlation between sPD-L1 levels and cancer stage suggests that sPD-L1 increases may represent an aggressive characteristic rather than increased tumor load.

Our study revealed that high pretreatment serum sPD-L1 levels and low treatment response (less than PR) were independent prognostic factors for lower PFS. Additionally, serum sPD-L1 level was a strong predictor of treatment response, suggesting that sPD-L1 plays a key role in MM progression and chemotherapy resistance. The mechanisms by which elevated sPD-L1 levels contribute to poor prognosis in MM are not clear, but there are several possible explanations. For example, in addition to inhibiting tumor-specific CTLs, PD-L1 binding to PD-1 induces drug resistance in MM cells via the Akt signaling pathway [19]. Additionally, PD-1-induced resistance to anti-myeloma agents is reduced by the PI3K/AKT inhibitor LY294002 [19]. Further studies are needed to identify how PD1/PD-L1 signaling impacts MM prognosis.

Patients achieving CR have a much better prognoses, regardless of whether they are newly diagnosed or relapse/refractory patients [3, 25, 26]. In this study, we found that good treatment response (CR+PR) is a favorable prognostic factor in MM. Moreover, we found that the ORR was higher in patients treated with novel drug regimens than in those with older drug regimens (69.2% vs. 43.6%, *p* = 0.036). However, higher ORR failed to translate into a survival advantage in terms of OS. Clinical trials demonstrate that although novel drug regimens improve the ORR, most could not prolong the OS compared to older drug regimens [27, 28]. Therefore, although novel drugs like bortezomib and lenalidomide improve treatment efficacy, novel anti-myeloma drugs with different mechanisms may help improve long-term survival. Immune-based therapeutic strategies that target checkpoint signaling with PD-1- or PD-L1-blocking antibodies might both inhibit tumor cell growth and restore host immune function in MM.

Although our findings suggest that sPD-L1 levels influence MM prognosis, additional studies could provide stronger evidence. Our conclusions are limited due to the retrospective nature of this study, the diverse therapeutic regimens of the patients examined, and the lack of cytogenetic and molecular abnormality analyses for most patients. Future studies should be conducted in a larger sample of patients receiving uniform treatment to verify both the prognostic relevance of pretreatment sPD-L1 levels and the cut-off value of 2.783 ng/mL we used to define high sPD-L1.

In conclusion, this is the first study to demonstrate the relationship between serum sPD-L1 levels and MM prognosis, including treatment response and disease progression. Serum sPD-L1, which can be easily measured in clinical practice, may be an important independent prognostic factor for this disease. These results suggest a role for sPD-L1 in the pathogenesis of MM and offer new insight into potential therapeutic strategies.

## MATERIALS AND METHODS

### Patients

A total of 81 patients with symptomatic multiple myeloma were enrolled in our study, and all patients were treated in Sun Yat-sen University Cancer Center between January 2008 and December 2014. The inclusion criteria were as follows: (1) de novo symptomatic multiple myeloma; (2) patients were given at least 4 cycles of chemotherapy; (3) serum at diagnosis was available; (4) complete follow-up information. Sun Yat-sen University Cancer Center Research Ethics Board approved use of the data in this study, and written informed consent for use and publication of patients' medical information was obtained from all patients at their first visit.

### Treatments and response evaluation

According to the patients' economic situation, novel drug-based (mainly bortezomib) or older drug-based (mainly anthracylines or melphalan) regimens were used (because bortezomib and lenalidomide are not covered by Chinese medical insurance). In total, 55 patients received older drug regimens, such as VAD (vincristine, adriamycin, and dexamethasone) [29], DVD (doxil, vincristine, and dexamethasone) [29], and MP (melphalan and prednisone) [30]; 26 patients received novel drug regimens, such as VD (bortezomib and dexamethasone) [31], PAD (bortezomib, adriamycin, and dexamethasone) [28], VTD (bortezomib, thalidomide, and dexamethasone), and CyBorD (cyclophosphamide, bortezomib, and dexamethasone) [32]. Patients were given at least 4 cycles of treatment, followed by autologous stem cell transplantation (if eligible) or maintenance with thalidomide. Treatment responses were evaluated after each cycle according to the International Myeloma Working Group (IMWG) criteria [33].

### Soluble PD-L1 measurement

Serum was collected at diagnosis and before treatment from all 81 patients (male to female ratio: 7:2, median age: 59 (range: 22–80)) and from 15 healthy individuals (male to female ratio: 4:1, median age: 54 (range: 20–72)), and stored as 500 ml aliquots at −80°C. Soluble PD-L1 was measured using an enzyme-linked immunosorbent assay (PDCD1LG1 ELISA kit, USCN Life Science, catalogue: SEA788Hu) according to the manufacturer's instructions. The minimum detectable concentration of sPD-L1 was 0.057 ng/ml. Each sample was analyzed in duplicate. The intra-assay and inter-assay coefficients of variation were below 20 percent.

### Statistical analysis

Receiver operating characteristic (ROC) curve analysis was performed to determine the best cut-off value for sPD-L1 concentration that would classify patients as having a high risk of disease progression (using SPSS version 19 statistical software). In this ROC curve, the point with the maximum sensitivity and specificity was selected as the cut-off value. Correlations between sPD-L1 concentration and various clinicopathologic parameters were assessed using the Mann-Whitney *U*-test or the Wilcoxon-matched test, and a chi-squared test or Fisher's exact test was used for categorical values. Progression-free survival (PFS) was the time between the date of diagnosis and the date of disease progression or death and was determined at last follow-up visit. Overall survival (OS) was the time between the date of diagnosis and date of death from any cause and was determined at the last follow-up visit. PFS and OS were calculated by the Kaplan–Meier method, while a log-rank test was used for comparison. The prognostic factors of OS and PFS were analyzed by univariate analysis. Multivariate analysis was performed using the Cox proportional hazard model to compare significant factors from the univariate analysis. Hazard ratio and 95% confidence interval were calculated for all variables in the regression model. A two-sided *p*-value < 0.05 was considered statistically significant. SPSS version 19 statistical software was utilized.

## SUPPLEMENTARY FIGURE


