# Supplementary material for: Serum levels of soluble programmed death ligand 1 predict treatment response and progression free survival in multiple myeloma
Source: Oncotarget. 2015 Oct 16;6(38):41228–36. doi: 10.18632/oncotarget.5682 (PMC4747402; doi:10.18632/oncotarget.5682)
Supplement: Supplementary file 1 [file oncotarget-06-41228-s001.pdf]

SUPPLEMENTARY FIGURE

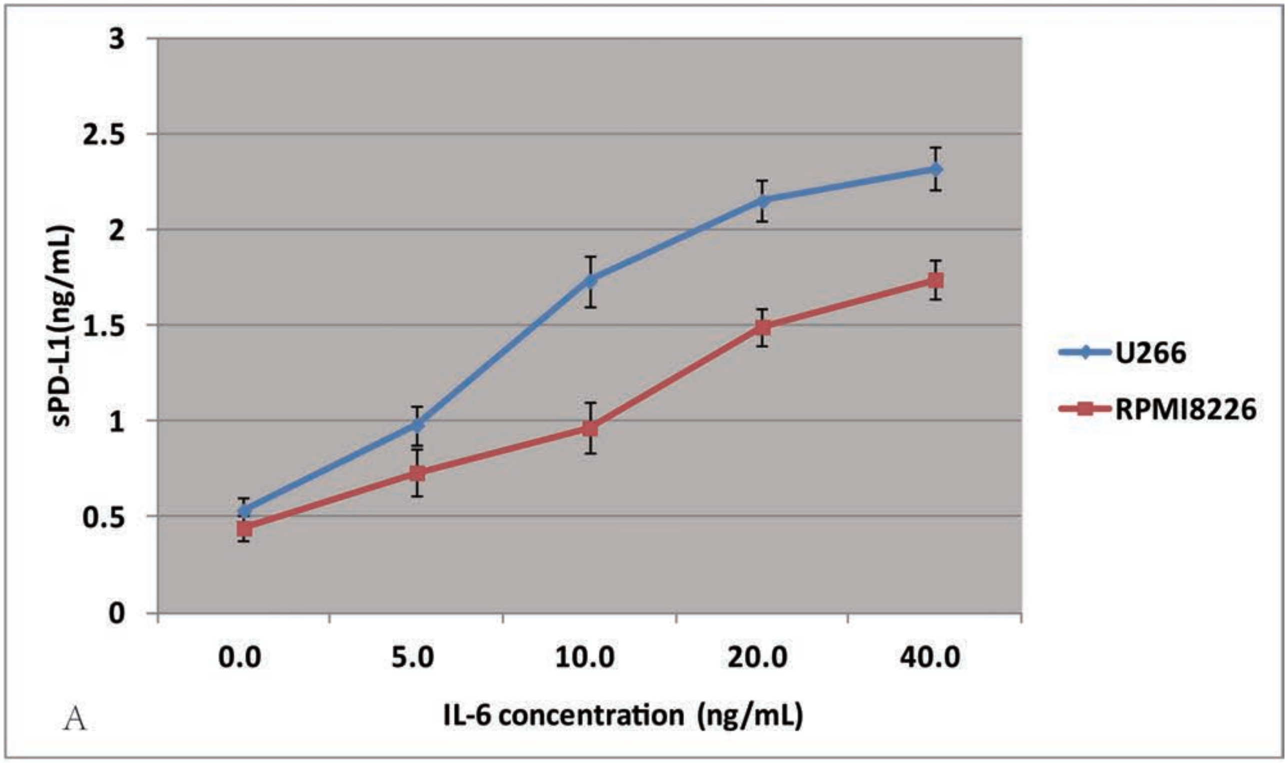

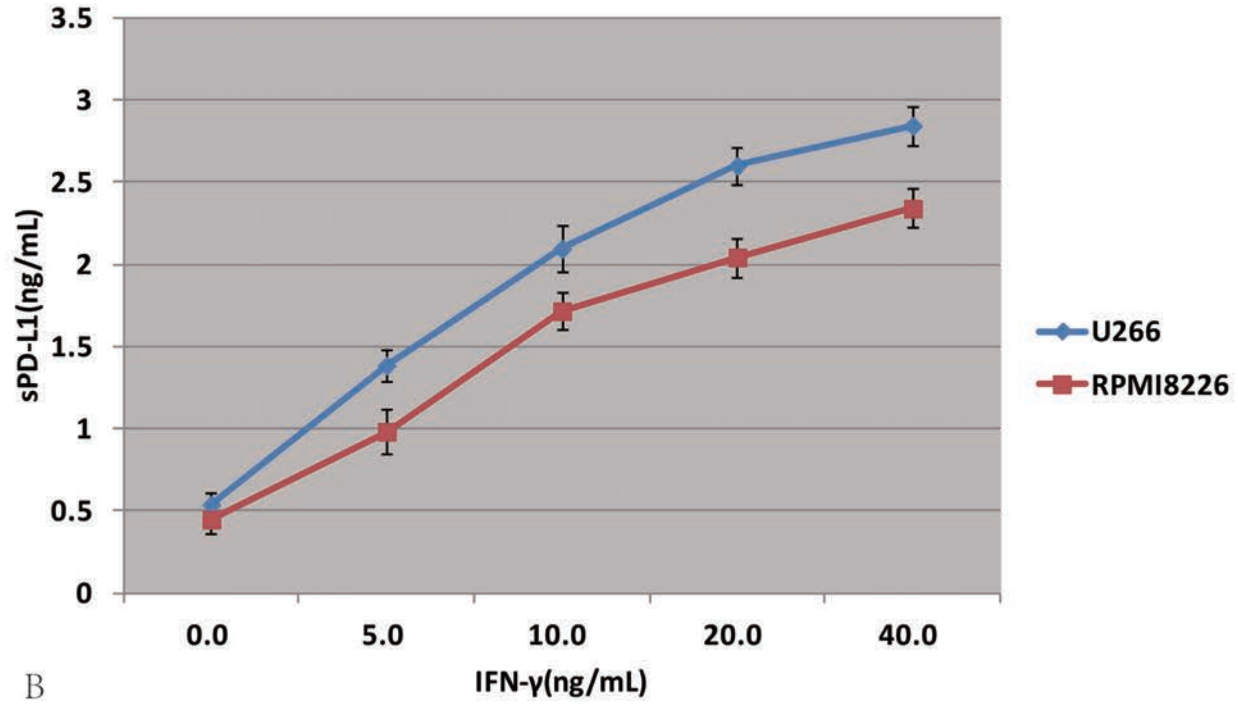

Supplementary Figure S1: Myeloma cell lines (U266 and RPMI8226) express sPD-L1 which can be induced by proinflammatory factors, such as IL-6 (A) and IFN-γ (B) with a concentration-dependent manner.
